# Perceived social support and presenteeism among healthcare workers in China: the mediating role of organizational commitment

**DOI:** 10.1186/s12199-019-0814-8

**Published:** 2019-09-04

**Authors:** Tianan Yang, Tengyang Ma, Pucong Liu, Yuanling Liu, Qian Chen, Yilun Guo, Shiyang Zhang, Jianwei Deng

**Affiliations:** 1 School of Management and Economics, Beijing Institute of Technology, Beijing, 100081 China; 2 Sustainable Development Research Institute for Economy and Society of Beijing, Beijing, 100081 China; 3 Chair of Sport and Health Management, School of Management, Technical University of Munich, Uptown Munich Campus D, Georg-Brauchle-Ring 60/62, 80992 Munich, Germany; 4 Human Resources Department, Guangdong Women’s and Children Hospital, Guangzhou, 510180 China; 5 Medical Affairs Department, Peking Union Medical College Hospital, Beijing, 100010 China; 6 Hospital Infection Management Department, The First Affiliated Hospital of Xiamen University, Xiamen, 361003 China

**Keywords:** Coworker support, Supervisor support, Organizational commitment, Presenteeism, Healthcare worker

## Abstract

**Objectives:**

We assessed the role of social support in presenteeism by examining organizational commitment among Chinese healthcare workers.

**Methods:**

One thousand four hundred thirty-four healthcare workers from 6 hospitals in 4 Chinese cities completed a questionnaire measuring presenteeism, social support, and organizational commitment. With organizational commitment as the mediator, regression analyses and structural equation modeling were used to test the model.

**Results:**

Organizational commitment was directly inversely associated with presenteeism (β = − 0.42, *p* < 0.001). Coworker support was moderately but significantly inversely associated with presenteeism (β = − 0.15, *p* < 0.001), but the path from supervisor support to presenteeism was not significant (β = 0.05, *p* > 0.05). The correlation between supervisor support and coworker support was significant (β = 0.71, *p* <0.001). Supervisor support and coworker support were significantly positively associated with organizational commitment (β = 0.41, *p* < 0.001, and β = 0.14, *p* < 0.001, respectively).

**Conclusions:**

Supervisor support was more important in promoting organizational commitment, while coworker support was more effective in reducing presenteeism. The mediating effect of organizational commitment was significant.

## Introduction

The concept of presenteeism has long attracted the interest of researchers. Presenteeism was first defined, in 1892, as attendance at work but with suboptimal performance [1–4]. Later, presenteeism came to include sickness presenteeism, a construct usually related to health problems [1, 5, 6]. More recently, the concept of presenteeism was extended to include negative conditions caused by physical and other events that reduce productivity in enterprises and organizations [1–4, 7, 8]. The enormous impact of presenteeism is difficult to measure. Recent studies reported that presenteeism was responsible for 3 and 1.8 times the financial burdens of medical illness and absenteeism, respectively [2, 3, 5].

Most research on presenteeism has focused on its predictors; mechanisms to address presenteeism have rarely been investigated. Johns divided the determinants of presenteeism into context (including social support, work interference, and work enhancement) and personal perspective (including work–life balance, job stress, and health) [1]; however, only social support interventions targeting presenteeism were reported to be effective in previous studies [1, 2, 7–9]. In some studies, social support was categorized as supervisor support (including supervisor attention to each worker through coaching, directing, helping fulfill workers job responsibilities, and performance evaluations) and coworker support (i.e., coworker willingness to assist others in completing work-related, service-based duties) [10–12]. These supports effectively address presenteeism because strong support enhances job satisfaction, performance, and productivity, and reduces presenteeism in organizations [7, 13, 14]. However, few subsequent empirical studies investigated potential interventions.

Social support should be enhanced in order to reduce workplace presenteeism. With respect to the relation between social support and presenteeism, only job stress, the most significant determinant of presenteeism [15], was examined as a mediator [7]. Additionally, organizational commitment—defined as the relative strength of an individual’s identification with and involvement in a particular organization [16]—significantly mediates the relationship between job stress and presenteeism [4]. No further mediator was identified in previous studies of social support and presenteeism. Interestingly, these two studies indicate that organizational commitment to the study of social support and presenteeism has a more direct influence on presenteeism than on work stress. Self-determination theory posits that people can be motivated by self-initiatives through both rewards and strengthening self-identification of value in organizations [17]. Social support as a kind of intrinsic motivation affects the internal self-worth of strong employees and strengthens organizational commitment [18], thus relieving presenteeism. To effectively address presenteeism, we analyzed organizational commitment as a specific mediator in our study.

**Fig. 1:**
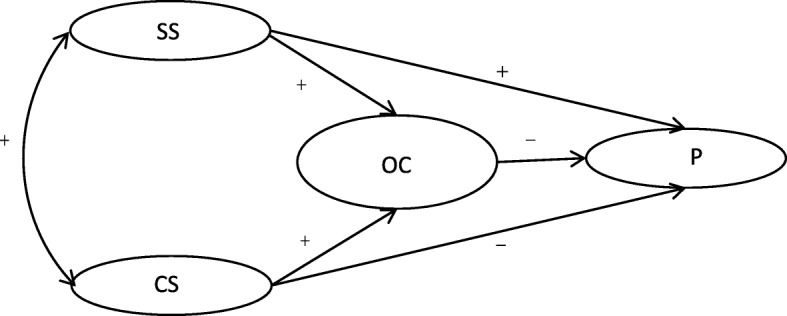
Proposed model of how supervisor support (SS), coworker support (CS), and organizational commitment (OC) affect presenteeism (P)

China is a policy-driven country, and the social effects of policies are much greater than in other countries [19–21]. In China, the primary concerns of medical reform are controlling medical costs and improving healthcare quality [20, 22]. Healthcare workers are required to provide an increasing number of healthcare services at a high level of quality. However, Chinese healthcare workers have been victims in numerous incidents of violence and face stricter qualification requirements and longer training periods as compared with other occupations. Occupational stress has resulted in poor physical and psychological well-being among healthcare workers [23–25]. Therefore, they have been unable to meet strategic requirements, which has limited healthcare efficiency in China [26–29]. In this study of the effectiveness of social support interventions for presenteeism, we investigated organizational commitment among Chinese healthcare workers (Fig. 1).

## Methods

### Data source

This cross-sectional study analyzed data from 1434 healthcare workers employed in class A tertiary hospitals in Guangzhou (479), Xiamen (251), Beijing (453), and Xinjiang (265) in 2016. Class A tertiary hospitals are classified according to the current “Hospital Classification Management Measures” and other provisions of the medical institutions [30]. It is the highest level in the classification of hospitals in China. Level 3 (more than 501 beds) is a regional hospital above which provides high level medical and health services and performs higher education and scientific research tasks in the areas and surrounding areas. Except for level 3, there are level 2 and level 1which are smaller than level 3. In level 3, grade A is a grade of hospitals that over 900 according to the classification criteria, and grade B is grade lower than 900 [30]. Ethics approval was received by an independent research ethics committee (including clinicians, nurses, administrative staff, medical technicians, and pharmacists). The survey assessed individual characteristics, perceived organizational support, organizational commitment, and presenteeism. To ensure data integrity and objectivity, participants were randomly selected by using employee numbers.

### Variables and instruments

Presenteeism was assessed with the 4-item perceived ability to work scale (PAWS), a reliable and valid instrument for measuring perceived productivity loss. This scale had acceptable psychometric properties in previous studies and in the Health and Retirement Survey in the USA study [31, 32]. The item, “Thinking about the mental demands of your job, how do you rate your current ability to meet those demands?” (Table 2), asks respondents to rate their perceived ability on a scale from 0 to 10 (0 = cannot currently work at all; 10 = work ability is currently at its lifetime best, Cronbach α = 0.865). To improve intuitive understanding of the score, we changed its directionality by subtracting the original scores from 10. Thus, higher scores indicate greater presenteeism.

Social support was measured with the three-item “co-worker support scale” and four-item “supervisor support scale” (5-point Likert scale, 1 = not helpful; 5 = strongly helpful, Cronbach α = 0.914) [33, 34]. Items 1 through 3 address coworker support, and items 4 through 7 address supervisor support. Higher values reflect greater support.

Organizational commitment was measured with 11-item organizational commitment scale (items 1 to 3) and career commitment scale (items 4 to 7) (5-point Likert scale, 1 = not concerned at all; 5 = greatly concerned, Cronbach α = 0.949) [35, 36]. Higher scores reflect greater concern for their organization.

### Statistical analysis

SPSS 20.0 and AMOS 21.0 were used for statistical analysis comprising descriptive analysis, analysis of means and standard deviations, subgroup analysis, and path analysis. Structural equation modeling (SEM) analysis was used to examine relationships among supervisor support, coworker support, organizational commitment, and presenteeism.

Before SEM, correlation analysis was used to determine the significance of correlations between supervisor support, coworker support, organizational commitment, and presenteeism. In SEM, four latent variables—presenteeism, supervisor support, coworker support, and organizational commitment—were first constructed by using the PAWS indicators, namely, the coworker support scale, supervisor support scale, and organizational commitment scale. The criteria used in evaluating the model were root mean square error of approximation less than 0.08 and goodness-of-fit, normed fit, comparative fit, and Tucker–Lewis index values of 0.90 or higher. All these indicators have been used to examine model fit in previous studies. The Sobel test was used to examine the effect of the mediator.

To determine if standardized regression coefficients (β) differed by subgroup, we conducted analyses of participants grouped by sex, education level, and job title. Education level and job title were both classified as lower and higher. Lower education level includes less than junior education, junior education, and bachelor’s degree. Higher education level includes master’s degree and doctor’s degree. Lower job title refers to primary and middle title while higher job title refers to deputy senior and senior title.

## Results

### Demographic characteristics of participants

Table 1 shows the demographic characteristics of healthcare workers. Among the 1434 participants, 32.7% were men and 67.3% were women; 29.8% were clinicians, 33.3% were nurses, 4.2% were administrative staff, 8.4% were medical technicians, and 2.4% were pharmacists. With respect to age group, 31.2% were 25–30 years of age, and only 5.2% were older than 50 years. With respect to education level, 42.1% had earned an undergraduate degree, 19.5% had earned a master’s degree and 13.7% had earned a doctorate. Half the respondents (49.9%) had a primary position, 30.8% had a middle position, and 15.5% were senior employees. Overall, 18.6% of participants had worked less than 3 years, 20.0% had worked 3–5 years, and 28.9% had work 6–10 years. Internal medicine (24.9%), surgery (14.3%), and obstetrics (12.2%) were the most common departmental affiliations; only 2.4% of participants were in the administration and logistics department (Table 1).

**Table 1:** Demographic characteristics of the participants

	Final sample (*n* = 1434)	Percentage (%)
Sex		
Male	457	32.7
Female	939	67.3
Age, years		
< 25	154	10.7
25~30	447	31.2
31~35	350	24.4
36~40	189	13.2
41~45	115	8.0
46~50	78	5.4
> 50	74	5.2
Position		
Clinician	428	29.8
Nurse	477	33.3
Administration staff	60	4.2
Medical technician	120	8.4
Chemist	35	2.4
Education		
Less than junior college	66	4.6
Junior college	263	18.3
Bachelor’s degree	604	42.1
Master’s degree	280	19.5
Doctorate	197	13.7
Title		
Primary	715	49.9
Middle	442	30.8
Deputy senior	155	10.8
Senior	68	4.7
Duration of employment, years		
< 3	267	18.6
3~5	287	20.0
6~10	414	28.9
11~20	271	18.9
> 20	170	11.9
Department		
Internal medicine	357	24.9
Surgery	205	14.3
Maternity	175	12.2
Pediatrics	126	8.8
Chinese medicine/rehabilitation	102	7.1
Emergency/intensive care unit	84	5.9
Infectious diseases/oncology	22	1.5
Other clinical departments	70	4.9
Medical technicians	127	8.9
Administration and logistics	34	2.4
Other	96	6.7

### Mean, SD, and correlations between presenteeism, social support, and organizational commitment

Table 2 shows the results (mean and SD) for the supervisor support, coworker support, organizational commitment, and presenteeism items. The means for the four presenteeism items were similarly low and ranged from 7.44 (“Rate ability to meet physical demands”; SD = 1.716) to 7.71 (“Rate current ability to work”; SD = 1.594). The means for the supervisor support items were lower than those for coworker support. The fourth supervisor support item (“My supervisor tries to make my job as interesting as possible”) had the lowest score (*M* = 3.47, SD = 0.915), and the first item had the highest score (*M* = 3.83, SD = 0.840). The first coworker support item had the lowest score (*M* = 3.84, SD = 0.727), and the third item had the highest score (*M* = 3.96, SD = 0.813). The means for the 11 organizational commitment items ranged from 3.52 (“I really care about the fate of this organization”, SD = 1.037) to 3.97 (“Satisfied ever entered nursing profession”, SD = 0.863).

**Table 2:** Mean and standard deviation (SD) for items related to supervisor support, coworker support, organizational commitment, and presenteeism

	Item	Mean	SD
Supervisor support (SS) (1–4)	1. My supervisor is helpful to me in getting the job done.	3.83	0.840
	2. My supervisor is willing to extend himself/herself to help me perform my job.	3.72	0.860
	3. My supervisor takes pride in my accomplishments at work.	3.57	0.848
	4. My supervisor tries to make my job as interesting as possible.	3.47	0.915
Coworker support (CS) (1–3)	1. My coworkers listen to me when I need to talk about work-related problems.	3.84	0.727
	2. My coworkers help me with difficult tasks.	3.87	0.725
	3. My coworkers help me in crisis situations at work.	3.96	0.813
Organizational commitment (OC) (1–11)	1. I really care about the fate of this organization.	3.97	0.863
	2. I am willing to put in a great deal of effort beyond what normally is expected in order to help this organization be successful.	3.73	0.924
	3. This organization really inspires me to put forth my best effort.	3.68	0.953
	4. Would not take other jobs paying same.	3.71	0.982
	5. Want career in nursing.	3.67	0.981
	6. If could do it all over, still choose nursing.	3.50	1.149
	7. If had all the money needed, still work in nursing.	3.60	1.048
	8. Ideal vocation too well to give it up.	3.59	1.030
	9. Ideal vocation for a life work.	3.56	1.045
	10. Satisfied ever entered nursing profession.	3.52	1.037
	11. Spend time reading nursing-related material.	3.74	0.907
Presenteeism (P) (1–4)	1. How many points would you give your current ability to work?	7.71	1.594
	2. Thinking about the physical demands of your job, how do you rate your current ability to meet those demands?	7.44	1.716
	3. Thinking about the mental demands of your job, how do you rate your current ability to meet those demands?	7.60	1.662
	4. Thinking about the interpersonal demands of your job, how do you rate your current ability to meet those demands?	7.59	1.616

Correlations between items are shown by the correlation coefficients(r) within the same construct (Table 3). Presenteeism was significantly inversely correlated with organizational commitment (*r* = − 0.43), coworker support (*r* = − 0.26), and supervisor support (*r* = − 0.26). Organizational commitment was significantly positively correlated with coworker support and supervisor support (*r* = 0.42–0.52). There was also a significant positive correlation between coworker support and supervisor support (*r* = 0.65).

**Table 3:** Intercorrelations between presenteeism (P), coworker support (CS), supervisor support (SS), and organizational commitment (OC) items

Variables (mean, SD)	Items			
	P	CS	SS	OC
P (2.41, 1.39)	1			
CS (3.89, 0.66)	− 0.26**	1		
SS (3.66, 0.78)	− 0.26**	0.65**	1	
OC (3.66, 0.81)	− 0.43**	0.42**	0.52**	1

*SS*, supervisor support; *CS*, coworker support; *OC*, organizational commitment; *P*, presenteeism

**p < 0.01

### Structural equation modeling

**Fig. 2:**
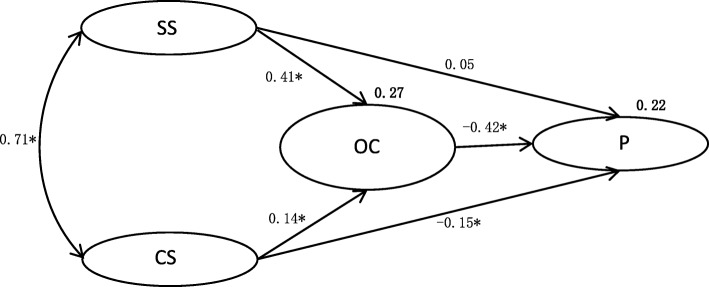
Final structural equation model, with standardized maximum likelihood estimates (*p < 0.001; numbers not in bold are standardized regression coefficients and numbers in bold explain variability)

In the SEM final model, organizational commitment was directly inversely associated with presenteeism (β = − 0.42, *p* <0.001). Coworker support was moderately but significantly inversely associated with presenteeism (β = − 0.15, *p* < 0.001), but the path from supervisor support to presenteeism was not significant (β = 0.05, *p* > 0.05) and was fully mediated by organizational commitment. There was a direct positive association between supervisor support and coworker support (β = 0.71, *p* < 0.001). Supervisor support and coworker support were significantly positively associated with organizational commitment (β = 0.41, *p* < 0.001, and β = 0.14, *p* < 0.001, respectively). Coworker support and supervisor support explained 27% of the variability in organizational commitment. Coworker and supervisor support and organizational commitment explained 22% of the variability in presenteeism. The revised model was more appropriate, as indicated by the root mean square error of approximation, goodness-of-fit index, comparative fit index, and normed fit index (Fig. 2).

We noted significant indirect effects between coworker support and presenteeism (Sobel *z* = −11.25; *p* < 0.001) and between supervisor support and presenteeism (Sobel *z* = − 12.09; *p* < 0.001), which were significantly mediated by organizational commitment.

Subgroup analyses (Table 4) showed that results of the model differed in relation to subgroup. For women and workers with less education, the path from supervisor support to presenteeism was significant (β = 0.11, *p* < 0.05, and β = − 0.21, *p* < 0.05, respectively). More interestingly, among workers with less education and less senior job titles, coworker support had no significant effect on organizational commitment (β = 0.07, *p* > 0.05, and β = − 0.6, *p* > 0.05, respectively).

**Table 4:** Standardized regression weights (β) with *p* values (α = 0.05) for the components of subgroup analyses

	Female		Male		Lower education level		Higher education level		Lower title		Higher title	Total		
	β	*p*	β	*p*	β	*p*	β	*p*	β	*p*	β	*p*	β	*p*
Path														
SS to OC	0.45	***	0.29	***	0.57	***	0.38	***	0.051	***	0.14	–	0.41	***
SS to P	0.11	*	− 0.05	–	0.21	*	0.02	–	0.06	–	− 0.03	–	0.05	–
CS to OC	0.11	*	0.25	***	0.07	–	0.18	***	0.06	–	0.32	**	0.14	***
CS to P	− 0.17	***	− 0.10	–	0.18	–	− 0.16	**	− 0.15	**	0.17	–	0.15	***
OC to P	− 0.44	***	− 0.38	***	0.51	***	0.39	***	− 0.41	***	− 0.43	***	0.42	***

*SS*, supervisor support; *CS*, coworker support; *OC*, organizational commitment; *P*, presenteeism

*Significant at 0.01 < *p* < 0.05

**Significant at 0.001 < *p* < 0.01

***Significant at *p* < 0.001. An en dash (–) indicates that the regression weight was constrained to 1.0 in the initial model

## Discussion

Coworker support had a significant inverse effect on presenteeism and slightly increased organizational commitment, while supervisor support was more effective in improving organizational commitment.

Our model can be explained by social exchange theory, which describes how people gain positive affect, trust, and kinship from outside. Blua maintained that social exchange occurs when one individual is attracted to another because the association is likely to be rewarding in some way, and because the interest in expected social rewards draws them to the other individual at work [37, 38]. When workers are satisfied, they develop feelings toward the organization, supervisor, and coworker, which are referred to as supervisor support and coworker support [39]. When workers feel such support, their trust in the organization is enhanced, which then manifests as organizational commitment. As part of the exchange, workers choose to behave better, and anti-productive behavior is reduced. In this way, we confirm the finding that organizational commitment can reduce presenteeism in the previous study.

The most valuable findings of this study are that coworker support adversely affects presenteeism, while supervisor support has no significant effect on presenteeism. Supervisor support has a stronger effect than coworker support on organizational commitment, because of differences between the roles of coworkers and supervisors. Regarding supervisor support, Mintzberg’s *The Nature of Managerial Work* holds that managers have 10 roles, including functioning as corporate spokespersons, information disseminators, and resource distributors [40]. Thus, managers represent the entire organization. A manager’s support of an employee means that the organization affirms the employee’s competencies and value in the organization, which can enhance his or her loyalty to the organization and sense of belonging, as well as the sense of organizational commitment [10, 16]. According to the theory of self-efficacy (i.e., whether a person can complete a job depends on a self-assessment of his/her own abilities) [41], promotion of organizational commitment ensures that employees feel they are important to the organization, which in turn stimulates initiative and reduces presenteeism and anti-productive behavior. Therefore, organizational commitment fully mediates the relationship between supervisor support and presenteeism.

With respect to coworker support, the job demand–resources model (JD-R) posits that the characteristics of any job can be divided into job demands and job resources. Job resources stimulate employee initiative to complete work goals (Jari 2008). Some researchers found that when the workload was large, coworker support as a job resource resulted in the sharing of tasks among employees, which relieved physical and psychological burdens and reduced presenteeism [42, 43]. Others noted that coworker support was linked to motivation and organizational commitment [43]. In the organization, relationships between colleagues are more relaxing and equal than those with supervisors. Employees can share their feelings with colleagues and benefit from their understanding [44]. The sympathy and understanding of colleagues can strengthen organizational commitment by making employees feel concern from the organization, thereby reducing anti-productive behavior [43]. The present results were consistent in part with those of previous studies [2, 3, 45, 46], which concluded that social support was important in promoting productivity, i.e., high social support was associated with greater productivity and less presenteeism [45–47]. The present study adds the valuable finding that supervisor support and coworker support have different mechanisms in relation to presenteeism. As noted in a previous study which found that organizational commitment was a more appropriate mediator in the relationship between social support and presenteeism. In future practice, enhanced supervisor support and coworker support for healthcare workers could be essential in reducing presenteeism [1]. In addition, increasing the sense of commitment among medical staff is important.

In subgroup analysis, the results for women, workers with less education and workers with senior jobs differed from those for the overall population. First, supervisor support has a direct positive effect on presenteeism among female employees but not among the overall population, because women are more perceptual while men are more reasonable [48]. Women care more about others, and medical institutions are expected to establish a multi-dimensional system to motivate female workers.

Second, supervisor support had a significant effect on presenteeism only among workers with less education. In addition, coworker support was not associated with organizational commitment or presenteeism in this subgroup. The JD-R suggests that supervisor support is more effective than coworker support at providing the resources needed for the repetitive work usually performed by less-educated employees [2, 3, 49]. Therefore, to improve social support at differing levels of job complexity, training in interpersonal relationship should be encouraged among Chinese public hospitals.

Among senior employees, supervisor support was not significantly associated with organizational commitment, and coworker support had no direct effect on presenteeism. This can also be explained by the JD-R model. Most senior workers are supervisors. They represent the entire organization, are more likely to give rather than receive support [49]. Supervisors value financial and human resources, as well as any opportunities to increase their social status. Thus, hospitals could encourage them by using these mechanisms.

Organizational commitment partially mediates the relationship between coworker support and presenteeism and fully mediates the relationship between supervisor support and presenteeism. This study examined organizational commitment as a mediating role was better than job stress [2]. In the present study, coworker and supervisor support explained 27% of the variability in organizational commitment, and coworker support, supervisor support, and organizational commitment explained 22% of the variability in presenteeism. A future study should focus more on the mediating effects of organizational commitment between job stress and presenteeism.

The present findings have potential theoretical and practical utility. First, the concept of presenteeism described by Hall concerned the relationship between presenteeism and health problems and considered health-related factors as the only indicators of presenteeism [27, 50, 51]. In the present study, we defined presenteeism as a behavior leading to productivity loss and found that presenteeism was related to health problems and psychological determinants such as organizational commitment, as indicated by the social exchange theory [1, 4, 24]. This finding may alter the conceptualization of presenteeism in future studies. Second, this study contributes to the reform of healthcare policy. Successful implementation of the Healthy China 2030 strategy requires that the well-being of healthcare workers be given top priority in a policy-driven country [28, 52]. More attention to job stress and presenteeism of healthcare workers would reduce productivity loss by providing appropriate support from leaders and colleagues. Furthermore, our findings indicate that supervisor and coworker support can enhance worker commitment to an organization and reduce productivity loss. Previous studies investigated several methods of increasing worker performance and reducing presenteeism, including improving the health conditions of workers [1, 2, 4, 8]. Future studies should examine methods of improving supervisor and coworker support.

## Limitations

This study has four limitations. First, the participants were mostly employees of Chinese class A tertiary hospitals, which limits the generalizability and accuracy of our conclusions. Second, our model examined only some of the determinants of organizational commitment. Third, this was a cross-sectional study; thus, our findings regarding presenteeism require confirmation in a cohort study. Fourth, the number of hospitals and research areas should be expanded in the future study and different types of Chinese hospitals would be useful.

## Conclusions

Chinese healthcare workers are becoming more important to policy-makers hoping to achieve the Healthy China 2030 goals. However, healthcare workers are exposed to considerable job stress and lack sufficient social support. This study found that to promote medical reform in China and cope with these challenges in Chinese hospitals, appropriate supervisor support and coworker support are critical in limiting presenteeism and sustaining high organizational commitment among healthcare workers.

## Data Availability

Please contact the corresponding author for data requests.

## Abbreviations

CS: Coworker support
JD-R: Job demand–resources model
M: Mean
OC: Organizational commitment
P: Presenteeism
SD: Standard deviation
SEM: Structural equation modeling
SS: Supervisor support
